# Verses

**Published:** 1907-09

**Authors:** 


### VERSES.

### SURE TO FOLLOW.

Turn about's fair play, methinks;
For instance when
Men set up the drinks, the drinks
Upset the men.

—Philadelphia Press.

### TIME WASTED.

Some men loaf till they grow old
And then they brag and blow
About the fortune they'd have made
Could they have had a show.

—Newspaper.

### AUGUST BARGAINS.

Now on the bargain counter go
The hats made out of straw,
And outing suits and bathing suits
More bargain hunters draw;
Which proves that good old summer-time
Has not much left of it,
And that would-be vacationists
Must now "get up and git."

—Exchange.

### RIDDLE.

You need me when the dark comes on.
And yet you cannot find me then.
I am a game, and yet I am
Of grave importance to most men.
When the same I am the best;
When opposite, the happiest.

—Exchange.

### THE GNATS.

I kin stan de flyin flies,
Kin scare em fum whar deys at,
But wat maks mer dander rise,
Is dem buzzin, pestrin, doggon gnats.

De skeeters cums long wid er tune
An dey bites like claws of cats,
An dey's after yer too fum moon ter moon,
But de cussedes tings is dem doggon gnats.

Dey gits in shirt, ears, eys an har,
An yer kaint ketch em no bettern rats.
So ob all de flyin pesses on yerth, hyar,
Durn dem confoundid doggon gnats.

B. H. Teague.

